# A novel *Babesia orientalis* 135-kilodalton spherical body protein like: identification of its secretion into cytoplasm of infected erythrocytes

**DOI:** 10.1186/s13071-018-2795-7

**Published:** 2018-03-27

**Authors:** Jiaying Guo, Jinfang Hu, Yali Sun, Long Yu, Junwei He, Pei He, Zheng Nie, Muxiao Li, Xueyan Zhan, Yangnan Zhao, Xiaoying Luo, Junlong Liu, Lan He, Junlong Zhao

**Affiliations:** 1 State Key Laboratory of Agricultural Microbiology, College of Veterinary Medicine, Huazhong Agricultural University, Wuhan, 430070 Hubei China; 2 Key Laboratory of Animal Epidemical Disease and Infectious Zoonoses, Ministry of Agriculture, Huazhong Agricultural University, Wuhan, 430070 Hubei China; 3 Key Laboratory of Preventive Veterinary Medicine in Hubei Province, Wuhan, 430070 Hubei China; 4 State Key Laboratory of Veterinary Etiological Biology, Key Laboratory of Veterinary Parasitology of Gansu Province, Lanzhou Veterinary Research Institute, Chinese Academy of Agricultural Science, Xujiaping 1, Lanzhou, 730046 Gansu China; 5 Guangdong Laboratory Animals Monitoring Institute, Guangdong Key Laboratory of Laboratory Animals, Guangzhou, 510663 Guangdong China

**Keywords:** *Babesia orientalis*, Spherical body protein, Erythrocyte, Localization, Cytoplasm

## Abstract

**Background:**

The spherical body is a distinct organelle only existing in *Babesia* and *Theileria*. Spherical body proteins (SBPs) are secreted from spherical bodies and incorporated into the cytoplasm of infected erythrocytes during invasion and post-invasion stages. Four different SBP homologues (SBP1, SBP2, SBP3 and SBP4) have been identified in *Babesia bovis* and *Babesia bigemina*. So far, there has been no report available about the identification of SBPs in *Babesia orientalis*.

**Methods:**

The SBP3-like in *B. orientalis* (BoSBP3-like) was cloned, sequenced, characterized and compared to the SBP3 sequences of *B. bovis* and *B. bigemina* by bioinformatics analyses. The BoSBP3-like gene was truncated into three fragments: BoSBP3-like-1 (915 bp), BoSBP3-like-2 (1311 bp) and BoSBP3-like-3 (1011 bp), which were amplified and cloned into the expression vector pET-28a and expressed as three truncated recombinant (His-fusion) proteins. The immunogenicity, native forms and localization of BoSBP3-like were identified by western blot and indirect immunofluorescence assay (IFA).

**Results:**

The BoSBP3-like gene was intronless with an open reading frame (ORF) of 3237 bp, encoded a 1079 amino acid polypeptide with a predicted size of 135 kDa, and contained a cysteine-rich region, three dispersing FAINT domains and a signal peptide (1–16 aa) at the N-terminus. The amino acid sequence of BoSBP3-like was 61.6 and 35.0% identical to that of *B. bovis* and *B. bigemina*, respectively. BoSBP3-like was identified as 135 kDa in the parasite lysate by rabbit antiserum against the truncated recombinant BoSBP3-like-1 (rBoSBP3-like-1). Three specific bands corresponding to rBoSBP3-like-1 (1–305 aa, 43 kDa), rBoSBP3-like-2 (306–742 aa, 58 kDa) and rBoSBP3-like-3 (743–1079 aa, 52 kDa) were detected by reaction with serum from *B. orientalis-*infected buffalo. The BoSBP3-like was not only localized in the spherical body of *B. orientalis* but also in the cytoplasm of infected erythrocytes of buffalo as puncta-like protein specks at both single and paired parasite development stages.

**Conclusions:**

Through secretion into the cytoplasm of infected erythrocytes, BoSBP3-like may play a significant role in adaptation, interaction, and modification related to the host environment to benefit the growth and survival of *Babesia*. BoSBP3-like could react with the serum from *B. orientalis*-infected buffalo, but not healthy buffalo, implicating that BoSBP3-like is highly antigenic and may serve as a candidate diagnostic antigen for the detection of *B. orientalis*.

## Background

*Babesia* spp. are tick-borne haemoprotozoan parasites and taxonomically classified in the phylum Apicomplexa, order Piroplasmida. *Babesia* spp. can invade a broad range of vertebrate hosts and even humans, causing serious problems in the livestock industry, pet animal health, wildlife protection and human public health throughout tropical and subtropical regions of the world [1, 2]. *Babesia orientalis* has recently been described based on data concerning parasite's morphology, transmission, pathogenicity and phylogenetic relationships. This parasite is the causative agent of one of the most important diseases of buffalo in central and southern China, characterized by fever, anemia, icterus, hemoglobinuria and even mortality, leading to immense economic losses [3].

The apical complex, the characteristic fine structure for apicomplexan parasites, consists of three specialized secretory organelles, rhoptries, micronemes and dense granules [4] and will release and incorporate proteins into the host environment during invasion and post-invasion [5, 6]. However, dense granules have not been identified in *Babesia* and *Theileria*. There is a unique organelle known as spherical body present in the apical complex of *Babesia* spp. and *Theileria* spp. which is a homologue to dense granules [6]. One to four relatively large spherical bodies within each merozoite are located subjacent to the rhoptries and the micronemes [7]. Several spherical body specific proteins (SBP1, SBP2, SBP3 and SBP4) have been only identified and characterized in *Babesia bovis* and *Babesia bigemina* [6–10]. All of these spherical body proteins are found localized either in the cytoplasm or cytoplasmic face of the infected red blood cells (iRBC). Similarly, dense granules were characterized to be localized either in the cytoplasmic face of iRBC or within a parasitophorous vacuole (PV). Dense granules were reported to involve in interaction with the host, stabilizing the parasite environment, disrupting infected erythrocytes and triggering host immune responses [11–14]. Despite so many differences between spherical bodies and dense granules, the released proteins may play the same roles during parasite invasion, nutrient intake, waste elimination and adaptation to the host environment.

To date, many efforts have been made to identify SBPs in *Babesia* and the mechanisms for their interaction with the host, but little information is available in this respect. In this study, we identified and characterized a 135 kDa spherical body protein 3-like of *B. orientalis* (BoSBP3-like). The structure, immunogenicity and localization of BoSBP3-like were investigated by western blot and IFA and compared with those of *B. bovis* and *B. bigemina.* Integrated results revealed BoSBP3-like as a candidate diagnostic antigen for detection of *B. orientalis* and a vaccine candidate antigen for further related research.

## Methods

### Experimental animals

Three buffalo (1 year old) were purchased from a *Babesia*-free area, and were confirmed to be free of *Babesia* and *Theileria* by microscopic examination and reverse line blot [15]. Two buffalo were splenectomized two weeks before intrajugular injection of 4 ml of *B. orientalis* (Wuhan Strain)-infected blood with 1% of parasitized erythrocyte, and the remaining buffalo was used as a control. Blood samples from the two buffalo were collected every day to monitor the parasitaemia until it reached 3%.

Nine Japanese white specific pathogen free (SPF) rabbits (2.5 kg each) were used for preparation of immune serum against recombinant BoSBP3-like-1 (rBoSBP3-like-1), recombinant BoSBP3-like-2 (rBoSBP3-like-2) and recombinant BoSBP3-like-3 (rBoSBP3-like-3), and one Japanese white rabbit was used as a control.

### Parasite and merozoite antigen

*Babesia orientalis* merozoite antigen was prepared as previously described with some modifications [16]. Briefly, the iRBC pellets were washed by phosphate buffer saline (PBS) and then lysed by Tris/EDTA/NaCl (TEN) buffer. The soluble antigen was extracted by centrifugation at 16,300× *g* for 30 min and washed twice in PBS to a final volume of 50–100 ml, then stored at -20 °C for further use.

### Genomic DNA, RNA extraction and complementary DNA preparation

The leukocytes of *B. orientalis-*infected blood were removed by using Plasmodipur Filters (EuroProxima, Arnhem, the Netherlands). Total RNA was extracted from 400 μl of the *B. orientalis-*infected blood using a TRIzol®RNA extraction kit (Invitrogen, Carlsbad, CA, USA) according to the manufacturer’s instructions. RNA was treated with DNase I (Invitrogen). A complementary DNA (cDNA) was amplified by reverse transcribed PCR (RT-PCR) using a FastQuant® RT Kit (Tiangen Biotech, Beijing, China) and stored at -80 °C until further analysis.

Genomic DNA (gDNA) was extracted from 200 μl of *B. orientalis*-infected blood by using TIANamp Genomic DNA Kit (Tiangen Biotech) by following the manufacturer’s instructions and then stored at -20 °C until further analysis.

### Cloning and sequencing of the truncated and full-length BoSBP3-like

The BoSBP3-like gene was amplified from both gDNA and cDNA by PCR using the specific primers (SBP3-F/R) listed in Table 1. The specific primers were designed based on genome sequence of *B. orientalis* [17]*.* The thermal cycling parameters included the activation of Taq polymerase at 95 °C for 5 min, 33 cycles of (denaturation at 94 °C for 30 s, annealing at 55 °C for 30 s, extension at 72 °C for 3 min), and a final extension of 10 min at 72 °C. The three truncated fragments of BoSBP3-like gene were amplified from gDNA using the same PCR procedure mentioned above with three different specific primers (SBP3-like-F1/R1, SBP3-like-F2/R2 and SBP3-like-F3/R3) listed in Table 1.

**Table 1:** Primers used for the subcloning and sequencing of recombinant plasmids

Primer	Primer sequence (5'-3')	Restriction endonuclease
SBP3-like-F	ATGAGGCGCTTCACCCTGCTAGCGTTG	
SBP3-like-R	TTACATGTCCATACCGACGCGGCATAG	
SBP3-like-F1	CGGATCCATGAGGCGCTTCACCCTG	*Bam*HI
SBP3-like-R1	GCGTCGACAATCTGAGTGAAGTTGACG	*Sal*I
SBP3-like-F2	GGAATTCATGGCCCCACCTTCTGCC	*Eco*RI
SBP3-like-R2	GCGTCGACTTACTGGAGGAAGAACTGG	*Sal*I
SBP3-like-F3	CGGATCCATGATCACTGCTTACGAC	*Eco*RI
SBP3-like-R3	GCGTCGACTTACATGTCCATACCGACG	*Sal* I

The amplicons were electrophoresed using 0.8% ethidium bromide-stained agarose gel and purified by EasyPure® Quick Gel Extraction Kit (Invitrogen). The purified DNA fragments were ligated into pMD19-T vector (TaKaRa Biotechnology, Beijing, China). The BoSBP3-like sequence and the absence of introns were confirmed by isolating and sequencing the positive plasmids (Tianyi Huiyuan Biological Technology, Wuhan, China).

### Bioinformatics analysis

The amino acid sequence of BoSBP3-like was translated using the ExPASY online tool (http://www.expasy.org/translate/). Protein sequences were analyzed by predicting the transmembrane (TM) regions using TMHMM 2.0 (http://www.cbs.dtu.dk/services/TMHMM-2.0/) and predicting the putative glycosylphosphatidy-l-inositol (GPI) anchors using GPI Prediction Server 3.0 (http://mendel.imp.ac.at/sat/gpi/gpi_server.html). BioEdit software was used to analyze the putative signal peptide (http://www.cds.dtu.dk/dervices/signalp/) and the potential subsequence motifs (http://mythis.isb-sib.ch/cgi-bin/motif_scan) in BoSBP3-like by combining the results of multiple sequence alignment among *B. orientalis*, *B. bovis* and *B. bigemina*. The secondary structure and the 3D-structure of BoSBP3-like were subsequently predicted by DNAstar software and I-TASSER standalone package, respectively [18–20].

### Truncated expression and purification of the recombinant proteins

The length of the BoSBP3-like gene is 3237 bp, which was too long for prokaryotic expression. The ORF of BoSBP3-like gene was truncated into three fragments: BoSBP3-like-1 (915 bp), BoSBP3-like-2 (1311 bp) and BoSBP3-like-3 (1011 bp), which were amplified and cloned into the expression vector pET-28a. The recombinant plasmids (pET-28a-BoSBP3-like-1, pET-28a-BoSBP3-like-2 and pET-28a-BoSBP3-like-3) were transformed separately into *E. coli* BL21 (DE3) strain. Small-scale culture was subjected to 1 mM isopropyl-β-D-thiogalactopyranoside (IPTG) induction overnight at 28 °C to identify the capacity and the forms of expression by SDS-PAGE analysis. BoSBP3-like-1/-2/-3 were expressed as His-fusion proteins, and purified using proteinPure Ni-NTA Resin (TransGen Biotech, Beijing, China) according to the manufacturer’s instructions. Briefly, the transformed *E. coli* was washed three times with PBS (PH = 7.4), lysed by ultrasonication in binding buffer containing phenylmethylsulfonyl fluoride (PMSF), and then centrifuged at 15,000×*g* for 15 min at 4 °C. Supernatants containing the rBoSBP3-like-1/-2/-3 were purified with the Ni-NTA Resin (TransGen Biotech).

### Preparation of polyclonal antibodies

Briefly, 500 μg of each purified rBoSBP3-like-1/-2/-3 in Freund’s complete adjuvant (FCA, Sigma, San Francisco, CA, USA) was subcutaneously injected into three Japanese white rabbits (specific pathogen-free, 3 kg each), respectively. Booster injections with the same antigen in FCA (Sigma) were given on days 14, 21 and 28. Fourteen days after the last immunization, serum from the immunized rabbits was collected and stored at -20 °C for further use.

### Western blot

For identifying the specific antigenicity of BoSBP3-like, rBoSBP3-like-1/-2/-3 were separated on 12% SDS-PAGE, blotted onto a nitrocellulose membrane (Merck, Kenny, NJ, USA) and blocked with 5% (w/v) skimmed milk in phosphate buffered solution Tween-20 (PBST). The membranes were probed with the serum (1:100 dilution) from the buffalo naturally infected with *B. orientalis* or with the serum from the negative control buffalo. Then, the membranes were incubated with BovIgG/HRP (1:2000) as secondary antibodies (Bioss, Beijing, China) and developed using the DAB method (ZSGB-BIO, Beijing, China) to identify whether the antibody response against rBoSBP3-like was elicited in *B. orientalis*-infected buffalo.

To determine the native BoSBP3-like in merozoites of *B. orientalis*, the anti-rBoSBP3-like-1/-2/-3 rabbit sera were prepared to react separately with the protein from the lysate of *B. orientalis*-infected buffalo erythrocytes and uninfected buffalo erythrocytes as follows: 0.5 mg (per lane) was subjected to 12% SDS-PAGE, transferred to a nitrocellulose membrane, and probed with the rabbit serum against rBoSBP3-like-1, rBoSBP3-like-2 and rBoSBP3-like-3 (1:100 dilution), respectively. As controls, lysate of *B. orientalis*-infected buffalo erythrocytes and uninfected buffalo erythrocytes were reacted with the serum of naïve rabbits. The membranes were washed with Tris buffered saline Tween-20 (TBST) and then incubated with goat anti-rabbit IgG/HRP (1:1000) as secondary antibodies (Bioss).

### Indirect immunofluorescence assay

For IFA, smears of *B. orientalis*-infected buffalo erythrocytes were prepared on slides, air-dried and then fixed in cold 50% acetone-50% methanol solution for 30 min at -20°C. The rabbit polyclonal anti-rBoSBP3-like-1/-2/-3 (1:200 dilution) and the naïve rabbit serum (as controls) were used as the first antibody on the fixed smears separately and incubated at 37 °C for 1 h. After washing four times with cold PBS, smears were incubated with goat anti-rabbit IgG conjugated to Alexa Fluor 594 (1:1000 dilution; Invitrogen) as the secondary antibody, followed by parasite nuclear staining using the DAPI stain (1:1000 dilution; Invitrogen). Another negative control consisted of the second antibody only without incubation with the primary antibody. Finally, the results were analyzed by fluorescence microscopy.

## Results

### Identification of BoSBP3-like gene

**Fig. 1:**
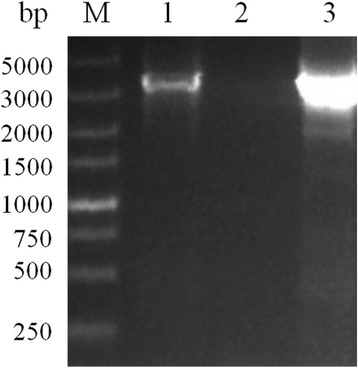
PCR amplification of the BoSBP3-like gene from *B. orientalis* cDNA and gDNA. Lane M: marker; Lane 1: amplicon from cDNA; Lane 3: amplicon from gDNA; and Lane 2: negative control

The BoSBP3-like gene was amplified from gDNA by using SBP3-like-F and SBP3-like-R primers (Table 1), and its full length was 3237 bp (Fig. 1). Sequence analysis indicated that BoSBP3-like encoded a polypeptide of 1079 aa, with a predicted size of 135 kDa. A 3237 bp fragment was obtained by cloning and sequencing the entire sequence coding for amino acids (CDS) of BoSBP3-like from gDNA and cDNA (Fig. 1). These results indicated that BoSBP3-like was intronless. The nucleotide sequence reported in this study has been submitted to GenBank with the accession number of KT947106.

### Bioinformatics analysis

The complete nucleotide sequence of BoSBP3-like revealed 70 and 76% similarity with the SBP3 gene of *B. bovis* (T2Bo) and *B. bigemina,* respectively. The amino acid sequences of SBP3-like were aligned using BioEdit software, and the results showed 61.6 and 35.0% identity to *B. bovis* SBP3 and *B. bigemina* SBP3, respectively. Additionally, the SBP3 genes of these three *Babesia* species showed high similarity at the C terminus.

The isoelectric point (IP) values of SBP3 of *B. orientalis*, *B. bovis* and *B. bigemina* were 6.495, 6.04 and 5.523, respectively. The high hydrophilicity and antigenic index suggested that all of them were good candidates for vaccine.

**Fig. 2:**
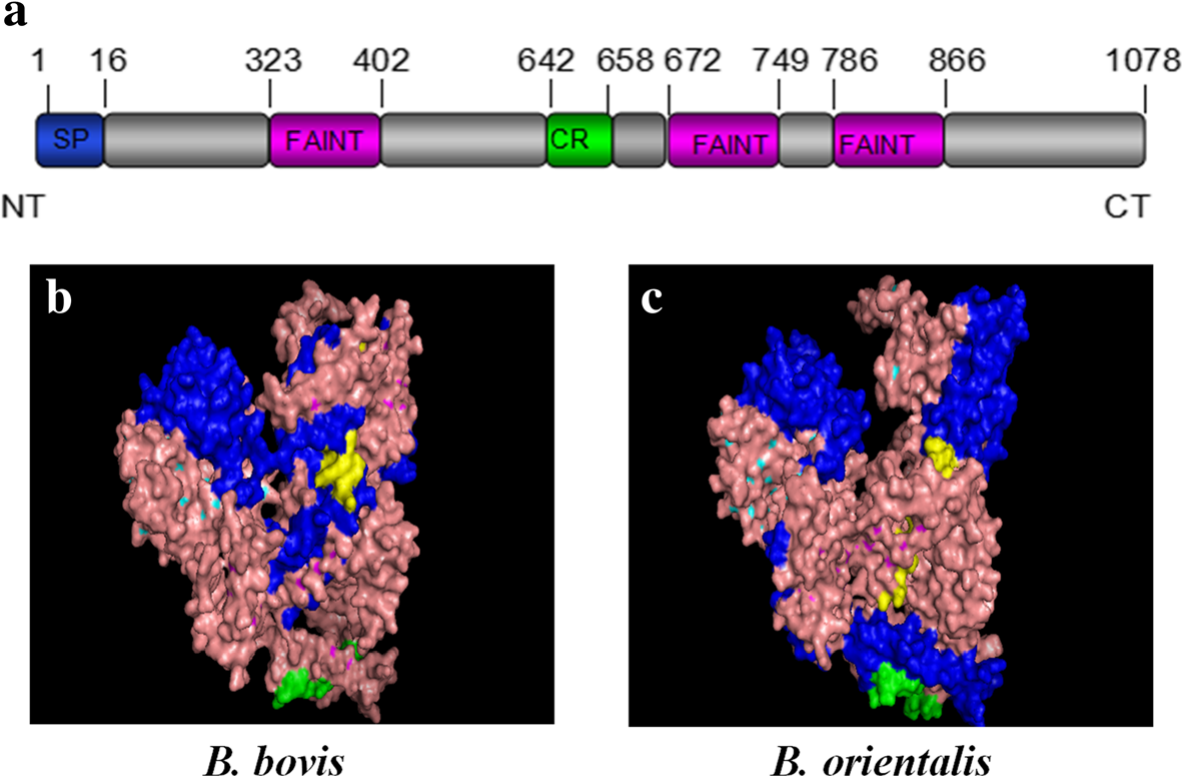
Schematic illustrations of predicted domains and crystal structures of SBP3. **a** Predicted domains of BoSBP3-like. A signal peptide (1–16 aa) at the N-terminus, three FAINT domains and a cysteine rich region. **b** Predicted crystal structure of SBP3 of *B. bovis* showing a signal peptide (yellow), three FAINT domains (blue) and one cysteine rich region (green). **c** The predicted crystal structure of BoSBP3-like

According to some bioinformatic criteria for identifying possible exported proteins, BoSBP3-like was predicted in TM, GPI and signal peptide. As shown in the results of TMHMM 2.0 and GPI Prediction Server 3.0, BoSBP3-like had no TM regions or any GPI anchors, which was consistent with SBPs in *B. bovis.* Following the similarity with SBPs in *B. bovis*, BoSBP3-like was revealed to contain a signal peptide (1 to 16 aa), a CYS-rich region and three FAINT domains. According to the predicted information, the motif of BoSBP3-like is shown in Fig 2. A comparison of the predicted 3D-structure of *B. orientalis* and *B. bovis* (Fig. 2) was also made to find the conserved regions and the specific domains in the surface and some NAG drug sites.

### Truncated cloning and expression of BoSBP3-like

**Fig. 3:**
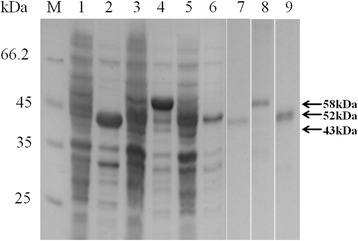
SDS-PAGE of expressed and purified rBoSBP3-like-1/-2/-3. Lane M: molecular weight marker; Lane 1, Lane 3 and Lane 5: lysate of un-induced plasmids pET-28a-BoSBP3-like-1/-2/-3; Lane 2, Lane 4 and Lane 6: lysate of IPTG induced pET-28a-BoSBP3-like-1/-2/-3; Lane 7, Lane 8 and Lane 9: purified rBoSBP3-like-1/-2/-3. The corresponding bands are indicated by arrows

According to the sequence analysis results, the BoSBP3-like gene was truncated into three fragments: BoSBP3-like-1 (915 bp), BoSBP3-like-2 (1311 bp) and BoSBP3-like-3 (1011 bp). The truncated fragments were expressed as His-fusion protein, and the sizes of rBoSBP3-like-1 (1–305 aa), rBoSBP3-like-2 (306–742 aa) and rBoSBP3-like-3 (743–1079 aa) were 43, 58 and 52 kDa, respectively. All rBoSBP3-like-1/-2/-3 were expressed in pellets. The rBoSBP3-like-1/-2/-3 were purified using Protein Pure Ni-NTA Resin (Fig. 3), then collected and dialyzed to immune rabbits.

### Identification of the recombinant and native BoSBP3-like

**Fig. 4:**
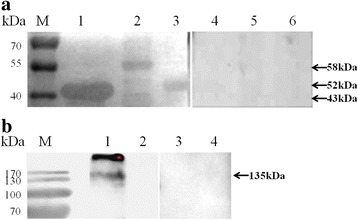
**a** Western blot analysis of BoSBP3-like immunogenicity. Lane M: molecular weight marker; Lane 1, Lane 2 and Lane 3: reaction of rBoSBP3-like-1/-2/-3 with the serum of *B. orientalis*-infected buffalo; Lane 4, Lane 5 and Lane 6: reaction of rBoSBP3-like-1/-2/-3 with the serum of normal buffalo. **b** Identification of native BoSBP3-like in *B. orientalis* merozoite lysate. Lane M: molecular weight marker; Lane 1: lysate of *B. orientalis*-infected buffalo erythrocytes probed with serum from a rabbit immunized with rBoSBP3-like-1; Lane 2: lysate of uninfected buffalo erythrocytes probed with serum from a rabbit immunized with rBoSBP3-like-1; Lane 3: lysate of *B. orientalis*-infected buffalo erythrocytes probed with serum of a naïve rabbit; Lane 4: lysate of uninfected buffalo erythrocytes probed with serum of a naïve rabbit. The corresponding bands are indicated by arrows

The specific antigenicity of BoSBP3-like was identified by western blot through the reaction of rBoSBP3-like-1/-2/-3 with the serum collected from the *B. orientalis*-infected buffalo, with the serum of the normal buffalo used as a control. Three specific bands corresponding to rBoSBP3-like-1 (43 kDa), rBoSBP3-like-2 (58 kDa) and rBoSBP3-like-3 (52 kDa) were detected in the serum from the *B. orientalis*-infected buffalo, but not in the serum of the control buffalo (Fig. 4a).

The native BoSBP3-like was also determined by western blot through the reaction of the anti-rBoSBP3-like-1/-2/-3 immune serum from the rabbits with the *B. orientalis*-infected buffalo erythrocytes, and the control buffalo erythrocytes. A band about 135 kDa was present in the *B. orientalis* lysate (Fig. 4b), which was consistent with the expected molecular weight of mature BoSBP3-like, but no band was observed in the negative controls. The other band may be attributed to the precursor of BoSBP3-like. The band of 135 kDa was detected by western blot only in the presence of anti-rBoSBP3-like-1 immune serum, rather than anti-rBoSBP3-like-2/-3 immune serum, indicating that the immunogenicity of rBoSBP3-like-1 may be better than rBoSBP3-like-2 and rBoSBP3-like-3.

### Localization of BoSBP3-like

The cellular localization of BoSBP3-like was determined using the polyclonal antibody generated from rabbits with the rBoSBP3-like-1/-2/-3. IFA was used to examine both the intracellular and extracellular parasite stages. However, the fluorescence of BoSBP3-like was only detected using rBoSBP3-like-1, rather than rBoSBP3-like-2/-3, which was consistent with the result of western blot in that only anti-rBoSBP3-like-1 immune serum from the rabbits reacted with the *B. orientalis*-infected buffalo erythrocytes.

**Fig. 5:**
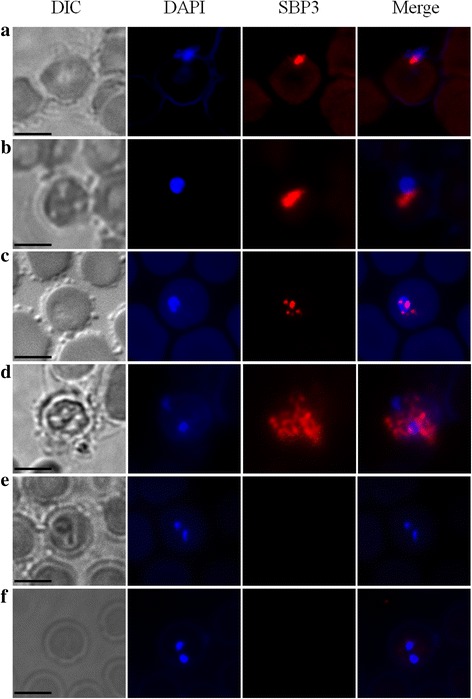
Localization and distribution of BoSBP3-like. Immunofluorescence and electron microscopy analysis of BoSBP3-like in the smears. Anti-BoSBP3-like serum (red) and nuclear staining of DAPI (blue). **a** Invasion stage. **b** The post-invasion stage of a single parasite. **c** At the post-invasion stage of a single parasite, BoSBP3-like was beginning to be secreted into the cytoplasm of iRBC. **d** At the post-invasion stage of paired parasites, BoSBP3-like was secreted into the cytoplasm of iRBC. **e** Negative control: the primary antibody was serum from a naïve rabbit. **f** Negative control: the second antibody only without incubation with the primary antibody. *Scale-bars*: **a**-**f**, 5 μm

In the extracellular parasite stage, fluorescence of BoSBP3-like was detected as puncta-like protein specks close to the nucleus of parasite. In the early intracellular parasite stage, BoSBP3-like was also detected as a puncta-like protein speck close to the nucleus of parasite. Then, the fluorescence of puncta-like protein specks of BoSBP3-like was detected throughout the cytoplasm of iRBC at both single and paired parasite development stages, while no fluorescence was observed in the uninfected RBCs and the other two negative controls. Despite the confirmation of the secretion of BoSBP3-like to the cytoplasm of iRBC, whether or not the secretion is on the cytoplasmic face of iRBC remains to be further elucidated (Fig. 5).

## Discussion

This study has identified and characterized a novel 135 kDa *B. orientalis* spherical body protein like, which shared a significant similarity to the SBP3 of *B. bovis*, and no critical amino acid identity to the other members of the SBP family (SBP1, SBP2 and SBP4). The significant similarity indicated that the identified gene was the BoSBP3-like gene. The BoSBP3-like was not only localized in the spherical body, but also in the cytoplasm of infected erythrocytes at both single and paired parasite development stages.

For elucidating the pathogenesis mechanism, there are two major processes for apicomplexan parasites: invasion and post-invasion. For the invasion stage, many efforts have been made to identify and characterize the groups of proteins released by apical complex, such as apical membrane antigen-1 (AMA-1), merozoite surface antigen (MSA), and Thrombospondin-related anonymous protein (TRAP) [21–23]. For the post-invasion stage however, little is known about the mechanism and related proteins involved in it. After invasion, iRBC will be altered in morphology and biochemistry. In morphology, iRBC will be more rigid, approximately three-fold higher than uninfected RBC in the mean value of the membrane shear elastic modulus [24], probably due to the presence of the non-deformable, abnormal intracellular parasite and modification of the RBC membrane skeleton proteins. On the other hand, the membrane of iRBC will be studded with ridge-like protrusion “ridges” which are described as knobs in *Plasmodium falciparum*. However in *Babesia*, ridges are found only present in *B. bovis*, but absent in *B. bigemina*, which may be related to pathogenicity [24]. In biochemistry, alterations in the iRBC adhesive properties occur in the adhesion of healthy uninfected RBCs and the number of other cell types, especially vascular endothelial cells, leading to sequestration and reduction of clearance by spleen. The reason for altered adhesive properties may be related to modifications of iRBC membrane components. Due to lack of protein synthesis pathways of differentiated erythrocytes, all of the modifications of membrane components will be derived from parasites. In *Plasmodium falciparum*, *Plasmodium falciparum* erythrocyte membrane protein-1 (PfEMP-1) and rifin have been identified and characterized as present on the face of iRBC [25]. While substantial efforts have been done to identify proteins on the surface of iRBC, no proteins have been identified to be expressed on the surface of iRBC in *Babesia*. Currently, *Babesia* SBPs have been characterized to be expressed at post-invasion and secreted to the cytoplasmic face of iRBC. Whether SBPs are involved into the formation of ridges needs to be further investigated.

Spherical bodies are members of the apical complex and only exist in *Babesia* and *Theileria*, which share a similar function with dense granules of *Plasmodium* and other apicomplexan organisms [2, 6, 7, 9]. Therefore, spherical body proteins may play a key role in host cell internalization. Notably, the parasites that contain dense granules all possess a parasitophorous vacuole (PV) during erythrocyte invasion, whereas PV will disintegrate after erythrocyte invasion in *Babesia* and *Theileria*, which contain spherical bodies but not dense granules [26]. These differences may indicate the possible relevance of disintegration of PV to spherical bodies. However, the association of disintegration of PV with spherical body proteins should be further investigated. In apicomplexan parasites, dense granule proteins participate in cell internalization and post-invasion process. For instance, ring-infected erythrocyte surface antigen (RESA) and ring membrane antigen (RIMA) are dense granule proteins of *Plasmodium falciparum*, which are released into the cytoplasm of iRBC and subsequently translocated to the cytoplasmic face of the iRBC [12]. Early immunoelectron microscopy and immunofluorescence studies showed that, in *Babesia*, only SBP1, SBP2 and SBP3 of *B. bovis* are secreted from spherical bodies, and subsequently released to the cytoplasmic face of the iRBC membrane at post-invasion [1, 6, 7, 27]. Previous studies on the localization of SBP4 in *B. bovis* demonstrated that SBP4 was secreted in a stage-dependent manner, which only happened during the early stage of single merozoites, the ring-form stage and the early cell-division stage [2, 10]. In this study, we identified the release of BoSBP3-like to the cytoplasm of iRBC for the first time. By indirect immunofluorescence assay, BoSBP3-like was detected not only close to the nucleus of the parasite but also throughout the cytoplasm of iRBC at both single and paired parasite development stages, demonstrating that BoSBP3-like was released into the cytoplasm of iRBC at all the parasite development stages. However, whether BoSBP3-like is released on the cytoplasm surface of iRBC has to be further studied. Meanwhile, a 135 kDa band was observed in the lysate of *B. orientalis* by western blot, indicating the existence of a novel antigen SBP3-like in *B. orientalis*. The other band may be attributed to the precursor of BoSBP3-like*,* which also needs to be further investigated.

In the present study, the BoSBP3-like gene was identified from both gDNA and cDNA as an intronless gene, comparatively like in a *B. bovis* homologue, sharing a nucleotide identity of 70% in the amino acid coding region [6]. Due to its significant similarity to the SBP3 of *B. bovis* and *B. bigemina*, we defined the identified gene as the BoSBP3-like gene. Comparative analyses of SBP3 amino acid sequences from *B. orientalis*, *B. bovis*, and *B. bigemina* revealed a relative conservation at the C terminus, indicating that SBP3 protein was conserved among *Babesia*. Additionally, BoSBP3-like was more close to *B. bovis* SBP3 in phylogenetic classification. Functional motif analysis revealed that SBP3 proteins were secreted into the host cytoplasm, and contained one to several FANIT domains. The FANIT domain was postulated to act as mediators of physiological changes associated with intracellular parasitism [10, 28]. Furthermore, SBP3 proteins in *B. orientalis* and *B. bovis* were revealed to contain a cysteine rich region, where structures were stabilized by disulfide bonds, which bypassed the more intensive selection needed to generate elaborate hydrogen-bonding interactions of complex α+β domain [29]. Previous studies have shown that cysteine-rich regions are involved in intermolecular or molecular interactions by mediating binding contacts with several residues due to the surface localization and the charged nature in cysteine rich domains [30]. In the present study, we found the signal peptide of SBP3 protein present in both *B. orientalis* and *B. bovis* at the N-terminus and displayed extracellularly, without TM regions or GPI anchors. This feature indicated that, as a secreted protein, BoSBP3-like processed a signal peptide that was necessary to guide the proteins into the secretory pathway and subsequently was exported to the infected erythrocyte. The absence of TM regions and GPI anchors meant that BoSBP3-like was not likely to be retained in the parasite. To understand whether there were any target sites in SBP proteins, we predicted and analyzed the 3D-structure of *B. orientalis* and *B. bovis*. Despite lack of available data on the full 3D-structure of the SBP3 protein, we constructed two predictive models using the I-TASSER software and x-ray diffraction in all existing protein databases. The combination of the predicted sequence characteristics, secondary structure motifs, partial domain localization in 3D-structure and similarity of SBP3 protein between *B. orientalis* and *B. bovis* led to the discovery of additional scientific information, which would facilitate further research on SBP3 protein functions. As illustrated, the signal peptide of the SBP3 protein in both *B. orientalis* and *B. bovis* was displayed at the N-terminus, which enabled this protein to be secreted and localized specifically. The cysteine-rich regions were also exposed at the same location, probably connected with their potential functions on molecular interactions. After protein 3D-structure prediction, NAG, a drug belonging to the anti-inflammatory category, was selected as the unique drug target based on the combined 3D-structure software prediction analysis of *B. bovis* and *B. orientalis*. Considering the involvement of BoSBP3-like in immune response, NAG component may be a potential effective drug for targeting BoSBP3-like.

In summary, we have identified and characterized a novel 135 kDa spherical body protein-like of *B. orientalis*, with a significant similarity to SBP3 in *B. bovis*. It is localized in the spherical body of *B. orientalis* and discharged to the cytoplasm of iRBC at all parasite development stages. BoSBP3-like can serve as a candidate antigen for detection of *B. orientalis*. These findings may facilitate a comprehensive understanding of SBP3 and further related research.

## Conclusions

The results of this article demonstrate that BoSBP3-like has a good immunogenicity and could act as a diagnostic antigen for *B. orientalis*. It was secreted into the cytoplasm of infected erythrocyte of buffalo at both single and paired shape parasite development stages, which indicates that BoSBP3-like might play a key role in post-invasion. This is probably the first report about identification and characterization of BoSBP3-like. However, the molecular mechanism of BoSBP3-like in the interaction with the host cell remains unclear and needs to be further investigated.

## Abbreviations

AMA-1: Apical membrane antigen-1
BoSBP3-like: The SBP3-like in *Babesia orientalis*
cDNA: Complementary DNA
CDS: Sequence coding for amino acids
gDNA: Genomic DNA
GPI: Glycosylphosphatidy-l-inositol
IFA: Indirect immunofluorescence assay
IP: Isoelectric point
IPTG: Isopropyl-β-D-thiogalactopyranoside
iRBC: Infected red blood cell
MSA: Merozoite surface antigen
NAG: N-acetyl glucosamine
ORF: Open reading frame
PBS: Phosphate buffer saline
PBST: Phosphate buffered solution Tween-20
PfEMP-1: *Plasmodium falciparum* erythrocyte membrane protein-1
PMSF: Phenylmethylsulfonyl fluoride
PV: Parasitophorous vacuole
rBoSBP3-like-1-2/-3: Recombinant BoSBP3-like-1/-2/-3
RESA: Ring-infected erythrocyte surface antigen
RIMA: Ring membrane antigen
RT-PCR: Reverse transcribed polymerase chain reaction
SBPs: Spherical body proteins
SPF: Specific pathogen free
TBST: Tris buffered saline Tween-20
TEN: Tris/EDTA/NaCl
TM: Transmembrane
TRAP: Thrombospondin-related anonymous protein
